# Rupture of an Industrial GFRP Composite Mitered Elbow Pipe

**DOI:** 10.3390/polym13091478

**Published:** 2021-05-03

**Authors:** Elsadig Mahdi Saad, Samer Gowid, John John Cabibihan

**Affiliations:** Department of Mechanical and Industrial Engineering, College of Engineering, Qatar University, Doha P.O. Box 2713, Qatar; samer@qu.edu.qa (S.G.); john.cabibihan@qu.edu.qa (J.J.C.)

**Keywords:** mitered elbow, GFRP, failure, water cooling pipeline

## Abstract

This paper examines the immature rupture of glass fiber reinforced plastic composite (GFRP) mitered elbow pipes. The GFRP composite mitered elbow pipe’s lifespan was twenty-five years; however, the pipes in question experienced immature failures, resulting in the reduction of their lifetimes to seven, nine, and ten years, respectively. The GFRP cooling water mitered elbow pipe’s service conditions operate at a pressure of up to 7 bar and temperatures between 15–36 °C. The root cause of failure was determined using visual inspection, analytical, microstructural, mechanical characterizations, and chemical analysis. The initial visualization inspection revealed an improper joint between the composite overwrapped and the straight pipe sections. Mechanical properties along the axial, hoop and 45° from the axial direction were obtained. The results from the analytical analysis indicated that the elbow might withstand the operating pressure depending on the quality factor, which was confirmed to be low due to the elbow joint’s improper fabrication process. As evidence of this, the numerical analyses’ results indicated that the safety factor in withstanding the operating pressure of 5 bar is dropped down in the radial region where the thickness is reduced to simulate the failure zone. This study’s findings recommend that thickness of less than 15 mm be reinforced using overwrapped composites. It is recommended for future installations that the fabrication process be appropriately monitored and controlled and avoids using 45°/−45° fiber orientation and multiple layers of chopped strand mat glass fiber.

## 1. Introduction

Glass fiber reinforced plastic composite (GFRP) pipeline is a lightweight, corrosion-resistant, and cost-competitive alternative for concrete, steel, and other plastic pipes, especially in large-diameter, moderate-pressure applications with good energy absorption and excellent bending behavior [1,2,3]. Nowadays, glass fiber pipes are gaining increasing importance in industry, as they are more durable and highly corrosion resistant. The use of glass fiber significantly eliminates the cost of cathodic protection needed with steel and reinforced concrete pipes in corrosive soils. Moreover, the glass fiber pipe weighs less than other pipe alternatives, which reduces the installation costs and increases the installation space. A glass fiber composite is a subset of Fiber-Reinforced Plastic (FRP) and includes a polymer (plastic) matrix and fibers for reinforcement. The fibers provide tensile strength, while the polymer resin (plastic) matrix provides structural rigidity (shape) and compressive strength. Several polymeric resins are used commercially in FRP pipes. Epoxy and polyester resins are commonly used in FRP pipes for domestic and irrigation water applications. Other resins include vinyl-ester and epoxy, which are more expensive and commonly used in applications where FRP pipes are exposed to highly corrosive liquids. Reinforcing fibers include glass fibers, carbon fibers, and aramid fibers, commonly used in the pipeline industry for oil and gas applications. However, fibers are susceptible to attack by chlorides and humidity; therefore, they are encapsulated in the polymeric matrix [4]. FRP is also used in numerous practical applications, such as fibrous porous media [5,6]. Pipeline designers use composite materials to eliminate the drawbacks of pipe welding, where two pipes are joined together [7]. Even though polymer matrix composites are utilized for weight reduction, their conventional manufacturing techniques, such as filament-winding and pultrusion, cannot meet the isotropic requirements [8]. Besides, nanomaterials and particles affect the properties and behavior of materials [9,10]. Researchers found a significant effect of fiber orientation on the composite pipes’ pressure capacity, and they attributed this effect to the composite materials’ anisotropic behavior under different loading directions. Being highly anisotropic, when loaded in the direction of the fiber orientation, composite materials are likely to exhibit the best mechanical properties [11,12]. If the fiber orientation is at ±45°, the generated strain due to service load is not principally in the axial and hoop direction. In this case, a maximum strain failure theory does allow cracks in the matrix before cracks occur in the fiber [13]. They used the axial and circumferential properties of the GFRP pipes using a set of tensile and compressive axial and circumferential tests. They found that the properties depend on the constituents and the volume fraction of each constituent. However, other researchers stated that each ply’s properties’ determinations are difficult due to the winding, interweaving patterns, the different chemical compositions of the constituents, and the curvature radius [14]. Researchers computed an excellent efficiency for the internally pressurized pipes based on the filament-wound glass fiber composite pipe’s mechanical performance. The results also revealed that using the Continuum Damage Mechanics (CDM) method in finite element simulations might lead to an internal pressure estimation error of as low as 7.4%. This error can be attributed to manufacturing inconsistencies. A steel pipe with a wall thickness loss defect was repaired using composite materials, where a bidirectional glass fibers fabric was wrapped on the outer surface of the pipe to repair the defect. The composite laminate was 300 mm wide and 16.2 mm thick. The experimental pressure tests proved that the composite repair’s efficiency failed at a pressure of 36.3 MPa [15]. Various investigations into the failure of composite-reinforced pipes have been carried out in the literature. Engineering failure analysis focuses on how a component or product fails during service or if a failure occurs due to the manufacturing process [16]. Structural integrity is related to both the material type and the structural geometry. Microscopically, material failures are related to crack initiation and crack propagation. Fatigue, environmental exposure, and aging lead to the failure of composite materials. The major types of fatigue failures are interlaminar tension and interlaminar shear [17]. Pipes are generally used to transfer fluids, pressured fluids, or highly corrosive products. Three-dimensional finite element stress and fracture analyses of a bonded socket joint reinforced with laminated FRP composite were conducted to study the influence of the internal pressure conditions on the stresses induced in a composite pipe. The fracture analysis revealed that the free edges of the adhered–adhesive interfaces are subject to adhesion failure under the influence of pressure [18]. The aging of a Glass-fiber Reinforced Epoxy (GRE) composite pipe used for seawater transportation was experimentally simulated and studied. The results suggested using GRE composite pipes for the prolonged flow of seawater for its low degradation rate. The modes of failure were identified as fiber pull-out, fiber–matrix debonding, pot-holing, and fiber breaking. Salt penetration reduced the moisture and induced pot-holing and fiber pull-out [19]. The failure of composite pipes that were subjected to external pressure was studied to investigate the ply stacking sequence’s effect on the composite pipe failure. The best stacking sequence under external pressure loading is the sequence that concentrates helicoidal layers closer to the neutral axis, followed by circumferential layers [20]. It was found that a sag water pipe took 12 years to finally fail due to the aging of the centrifugally cast GFRP composite relining. The hoop bending stresses were locally increased in the composite shell due to aging and the partially incomplete backfilling between the original pipe and the composite relining [21]. Stochastic failure analysis of a composite pipe subjected to a random excitation was conducted to study the effect of layup patterns on the pipes’ vibration and failure. It was concluded that the fiber angle affects the pipe’s natural frequency and that the pipe becomes weaker as the fiber winding angle increases. The safest layup patterns were found to be [902/±25]S and [902/±65]S, respectively. The notation [902/±25]S means two layers with a fiber orientation of 90 degrees, a layer with 55 degrees, and another layer with a fiber orientation of 115 degrees, given that the fiber orientation in each ply is measured with reference to the pipe longitudinal axis [22]. 

Despite the increasing importance of composite elbows (bent pipe), research activities on composite elbows design and fabrication are still scarce. The stresses and strains in bends manufactured by fiber-reinforced composites were introduced and analyzed in 1986. The results demonstrated the influence of geometry and fiber orientation on both the strength and the failure mode [23]. Two pipes connected by an elbow in the middle were analyzed in Abaqus under inner pressure loading. The study proved the effectiveness of the Hashin criterion [24]. The influence of the winding angle effect on the strength of composite materials and composite elbows was carefully studied [25,26,27,28] and the results demonstrated the accuracy of the geodesic method [29]. A double-bent composite elbow was manufactured and tested, and the results were compared to a finite element model that was solved using Simcenter software. The two elbow corners were the weakest in both the inner and the outer layers. The load capacity simulation error was 4.15%, while the deformation error was 7.75% [8]. In conclusion, the fracture of composite pipes has been thoroughly investigated in the literature; however, more studies are still needed to better understand fracture causes and behavior when pipes are utilized in different applications. It has been observed that the failures in mitered composite elbows have not been covered enough in the literature, and hence, this paper analyses the immature failure in an industrial composite mitered elbow and suggests improvements to the design.

## 2. Background

**Figure 1:**
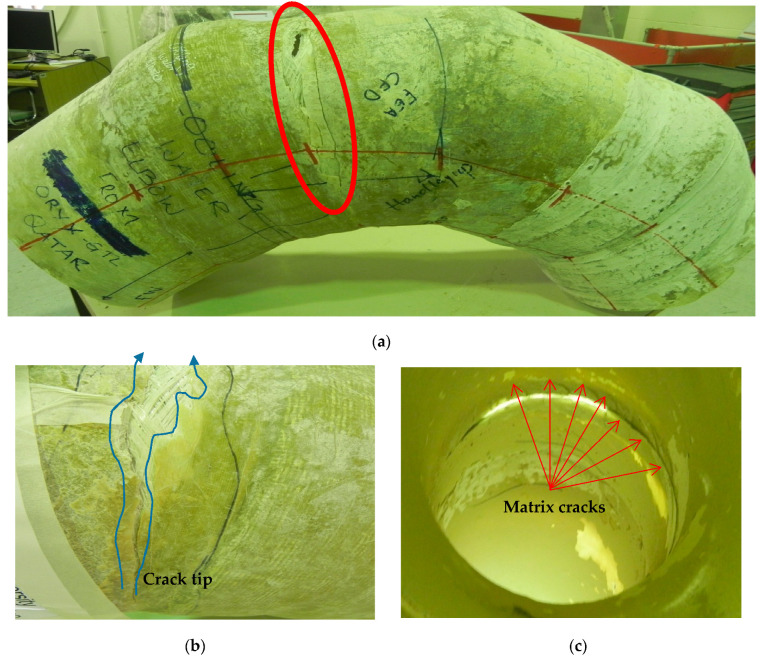
(**a**) Failed elbow; (**b**) a radial crack showing matrix cracking and fiber breakage; (**c**) transverse matrix cracks.

A GFRP cooling water mitered elbow pipe has experienced a structural failure at a ninety-degree mitered bend elbow, as shown in Figure 1a. The crack is circumferential on the elbow’s extrados approximately in the middle of the bend angle. The technical details are summarized below in Table 1. The pipe was reported to be S-glass fiber/polyester. Figure 1b shows the crack initiation and propagation, while Figure 1c shows transfer matrix cracks at the crack tip. Figure 1c shows the typically mitered elbow. The curing process was reported to follow the gelation stage, hardening stage, and maturation stage. The curing process took 12 h at a temperature between 32 °C and 47 °C. Table 1 also lists the mechanical properties of the polyester resin as provided by the supplier.

## 3. Initial Inspection

**Figure 2:**
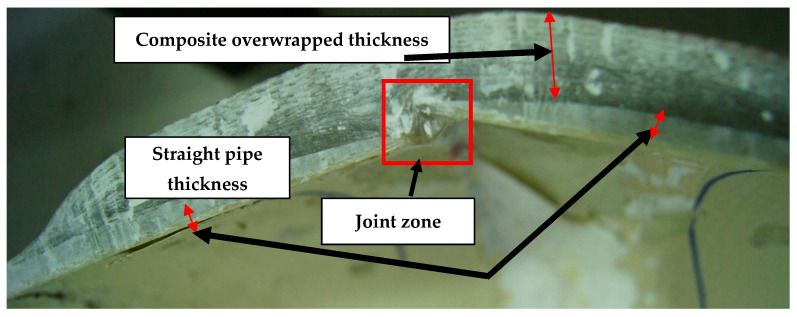
Elbow cross-section shows an improper joining between the composite elbow sections.

**Figure 3:**
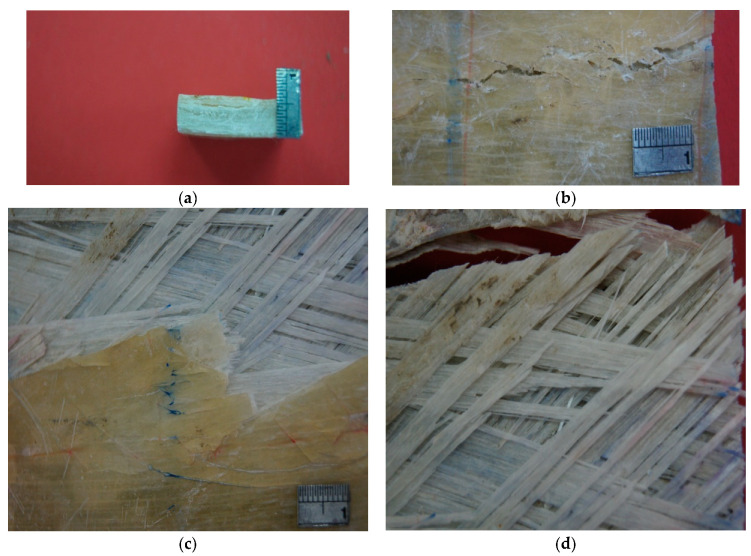
Fractured surface of (**a**) Interlaminar cracks throughout the elbow wall; (**b**) Interlaminar cracks at different magnifications; (**c**) Circumferential crack was observed to be initiated by matrix cracking; (**d**) Close up of crack revealing, matrix cracking, poor fiber–matrix interface, fiber debonding, and delamination at tip crack.

Initial inspection of the crack revealed that the fibers in the elbow pipe wall’s center were dry and very brittle (as seen in Figure 1b), indicating a poor fiber–matrix interface. When the component was cut in half longitudinally, it was also observed that the wall thickness varied significantly in the axial, radial, and hoop directions. An internal ridge was observed at the elbow entrance and exit, where the straight section was joined to the elbow. A visual analysis of the damaged composite proved that leaking results in through-thickness cracks within each ply, together with delamination, thereby forming a convoluted path through the pipe wall. An improper joint throughout the elbow was observed, as can be shown in Figure 2. Figure 3a–d show very poor bonding between the composite overwrapped and the straight pipe sections arranged to form the elbow section.

## 4. Chemical Analyses

The objectives of performing chemical analyses are identifying the elbow-joint material and finding some information about the leakage process. The chemical analyses were performed at three different locations using the X-Ray Spectrometer JSX-3201M Element Analyzer. The average of the results from the specimen’s chemical analysis and the elbow joint’s possible material are shown in Table 2. Chemical analyses of the elbow-joint material revealed it to be E-glass fiber instead of S-glass fiber. 

## 5. Sem Examination

**Figure 4:**
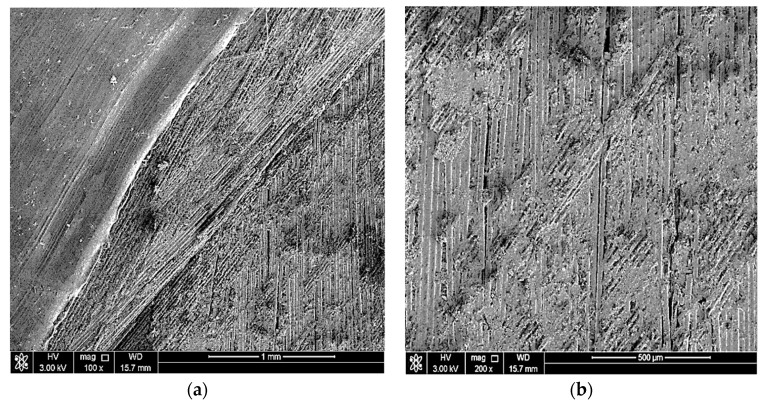
SEM images of the fractured region at (**a**) 100× and (**b**) 200×.

**Figure 5:**
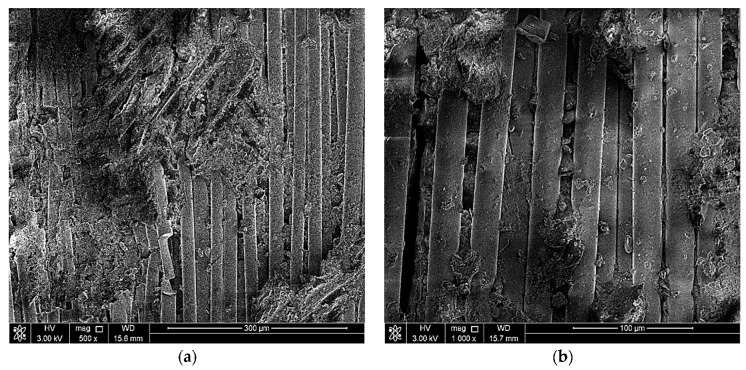
SEM images of the fractured region at (**a**) 500× and (**b**) 1000×.

**Figure 6:**
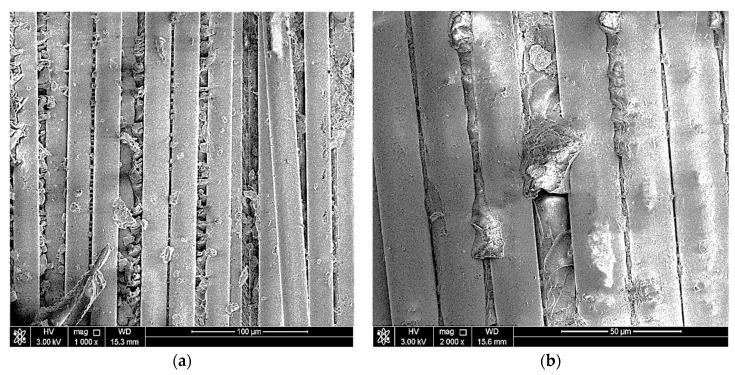
SEM images of the fractured region at (**a**) 1000× and (**b**) 2000×.

The objectives of conducting a scanning electron microscope (SEM) examination on the elbow-joint were to identify the surface’s condition, examine in more detail the cracks in the material, and spot defects in the material, if present. Two of the three parts were scanned at five magnifications (100X, 500X, 1000X, and 2000X) using the scanning electron microscope. Figure 4, Figure 5 and Figure 6 show the SEM results. The fractured surface was undulated with some bundles of loose and broken fibers (fiber bridging). The matrix fracture was quite brittle, exhibiting only brittle features fracture morphology in the matrix. At higher magnifications, such as 1000X and 2000X, the delamination between layers is very clear and indicates an improper fabrication process of the elbow-joint. Note that the matrix crack initiated the cracks, and with repeated loading, the crack eventually reached the surface and caused the reported leakage. 

## 6. Mechanical Testing

**Figure 7:**
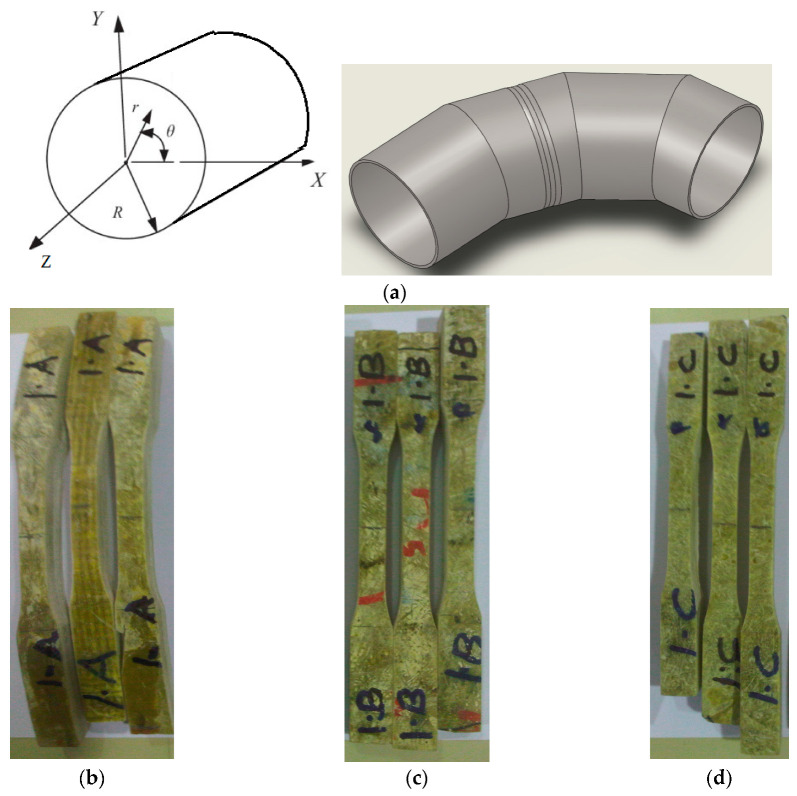
(**a**) The pipe section shows the coordinate system (r, θ, z); (**b**) Hoop specimens (along θ direction); (**c**) Axial specimens (along the z-direction); (**d**) 45° direction specimens.

**Figure 8:**
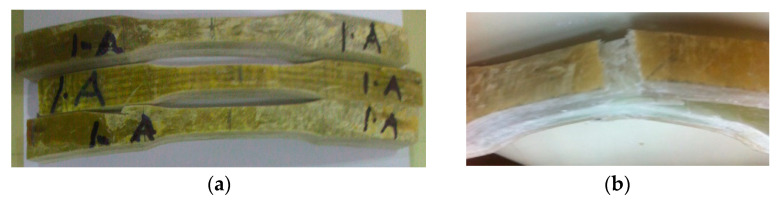

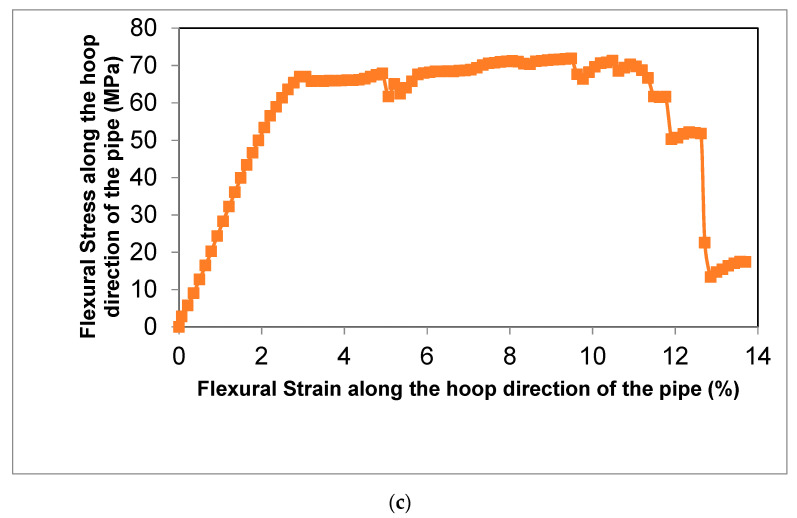
(**a**) Prepared specimens machined along the hoop direction; (**b**) Failed specimen showing matrix cracking, fiber debonding, and huge delamination between the straight pipe and the overwrapped fiber breakage; (**c**) Flexural stress–strain curve of hoop specimens.

**Figure 9:**
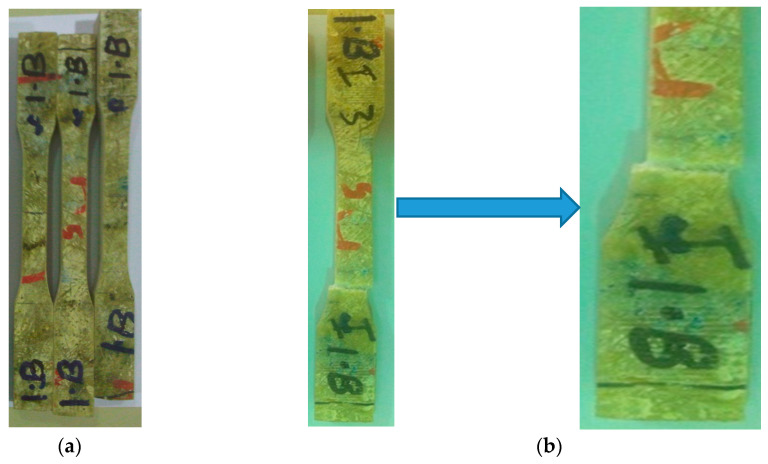

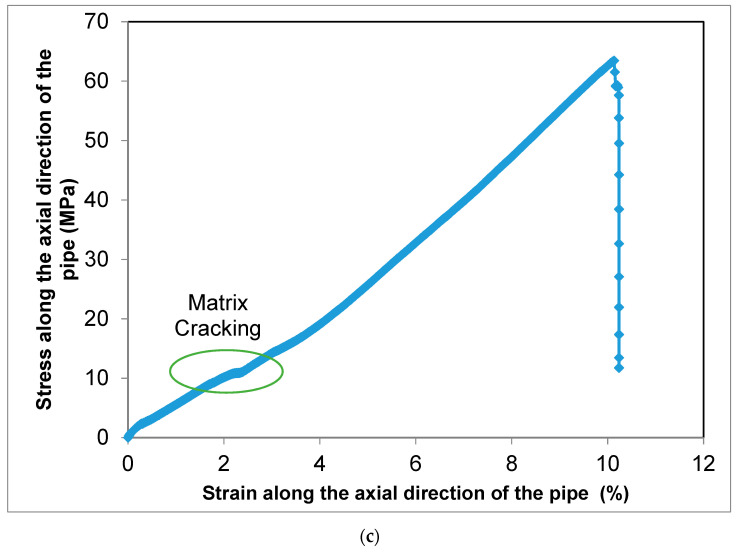
(**a**) Prepared specimens machined along the axial direction; (**b**) Failed specimen showing matrix cracking, fiber debonding, and huge delamination between the straight pipe and the overwrapped composite; (**c**) Stress–strain curve of axial specimens.

**Figure 10:**
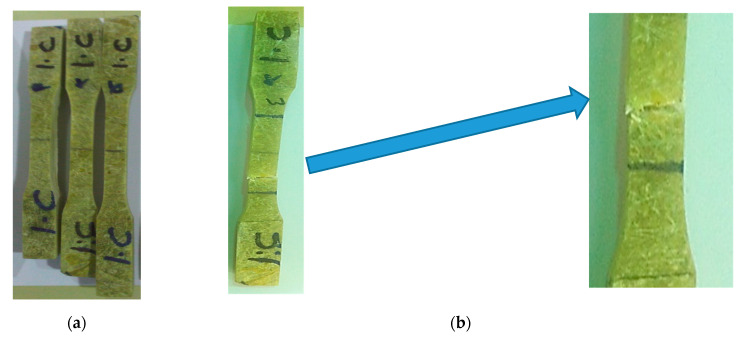

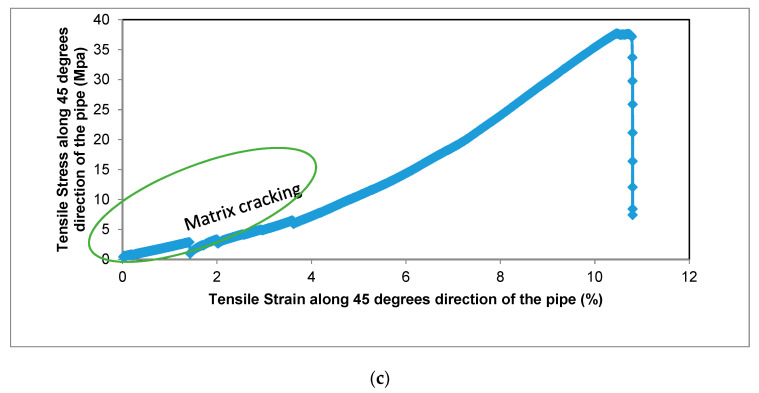
(**a**) Prepared specimens machined along the 45° direction; (**b**) Failed specimen showing matrix cracking and fiber breakage; (**c**) Stress–strain curve of 45° direction specimens.

**Figure 11:**
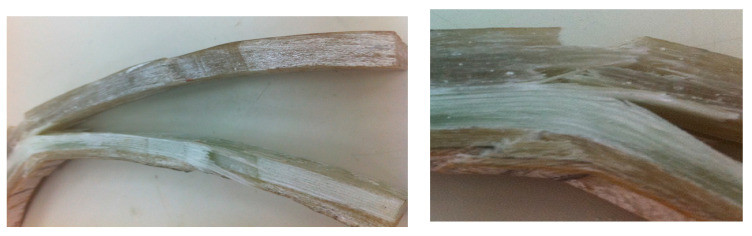
A severe delamination between the composite overwrapped and the joined straight pipes sections.

The elbow pipe was wound with a fiber orientation of ±45° and as the mechanical properties of the composite are direction-dependent, the generated strain due to service load was not principally in the axial and hoop direction. To obtain the mechanical properties along the axial, hoop, and 45° from the axial direction, nine specimens were machined from a different location, as shown in Figure 7. All mechanical testing was performed using an Instron 250 kN testing machine with a 0.01/min strain rate. The results are shown in Figure 8, Figure 9 and Figure 10. The tensile tests on the hoop specimens show an Ultimate Tensile Strength (UTS) of 60 MPa and a maximum strain of 8.7%. Figure 8a,b show the specimens before and after the tensile test. Figure 11 demonstrates the increase in the hoop specimens’ delamination area under tension and compression loading state. However, the axial direction specimen, as can be shown in Figure 9c, shows a critical point on the stress–strain graphs as highlighted.

A noticeable “knee” occurs in the stress–strain curves at approximately 10 MPa of stress and 1.7% strain. This irregularity is attributed to the formation of the first microcrack in the matrix. It is also due to the bond between the matrix and the fibers breakage on a microlevel, which is attributed to the resin system’s toughness and adhesive properties. This finding agrees with the flexure test results by [13]. As the line is located at 2.5 m depth, the water table is around 1.2 m near a coastal area; the microcracked laminate absorbs considerably more water than an uncracked laminate. The point labeled on the curve can be considered the point of failure of the component for two reasons. First, the crack becomes a weakness in the laminate exaggerated by the water pressure over time. Second, the microcracks allow the water to ingress into the laminate and allows its absorption by the matrix. However, in piping design, the pipe is considered to fail when it leaks. This damage weakens the laminates’ load-bearing capacity and accelerates the failure process. Since the stress profile is not uniformly distributed throughout the elbow, a failure in the layers occurs very locally. Therefore, the maximum allowable stress on the material for the operating conditions prescribed can be taken as 10 MPa (i.e., the point of the first microcrack occurrence) rather than the ultimate strength of 60 MPa. 

## 7. Analytical Analysis

**Figure 12:**
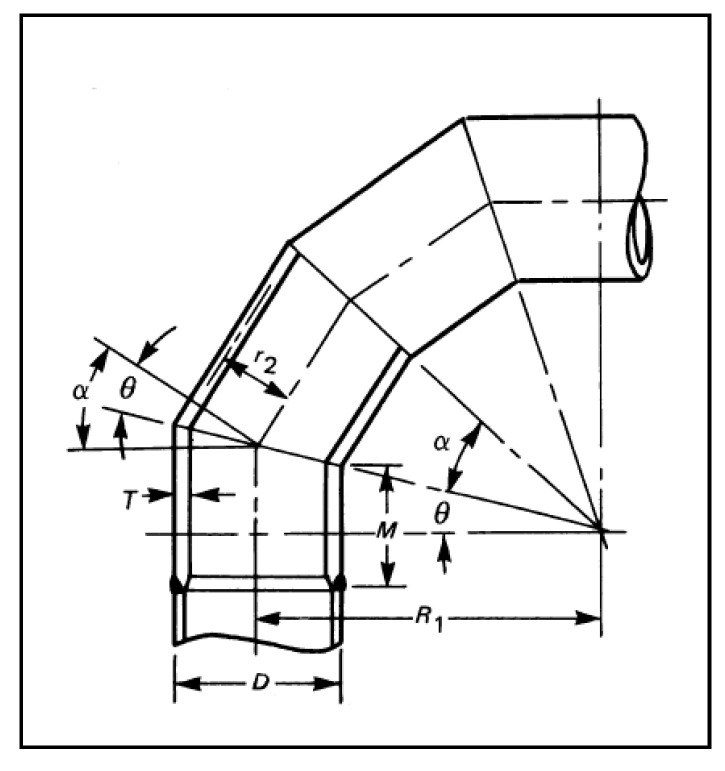
Mitered bend nomenclature as per ASME B31.3.

ASME code B31.3-2006 “Process Piping” includes the analytical formulae that can be used to determine the maximum operating pressure allowable for a miter bend. Figure 12 was extracted from ASME B31.3, and details the nomenclature for miter bends recommended by ASME. According to ASME B31.3, the maximum allowable internal pressure shall be the lesser value calculated from the following equations.

(1) $$P_{m}=\frac{SEW\left(T-c\right)}{r_{2}}\left(\frac{T-c}{\left(T-c\right)+0.634\;tan\theta \sqrt{r_{2}\left(T-c\right)}}\right)$$

(2) $$P_{m}=\frac{SEW\left(T-c\right)}{r_{2}}\left(\frac{R_{1}-r_{2}}{R_{1}-0.5r_{2}}\right)$$

where:

*S*: Material strength

*E*: Quality factor

*W*: Weld factor (N/A) = 1.

*T*: Wall thickness

*R*_1_: Bend radius

*r*_2_: Pipe radius

*c*: Allowance for threads or grooves (N/A) = 1

θ: miter angle

**Figure 13:**
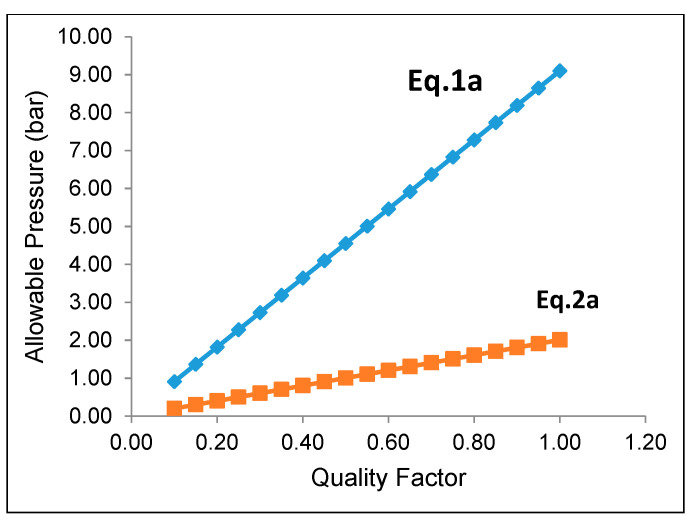
Allowable internal pressure as per ASME B31.3.

The value of “S” is taken as the value obtained by experimental testing (60 MPa). All geometric values were measured from the elbow and its virtual model. The quality factor “E” is varied, and the results are plotted below in Figure 13.

**Figure 14:**
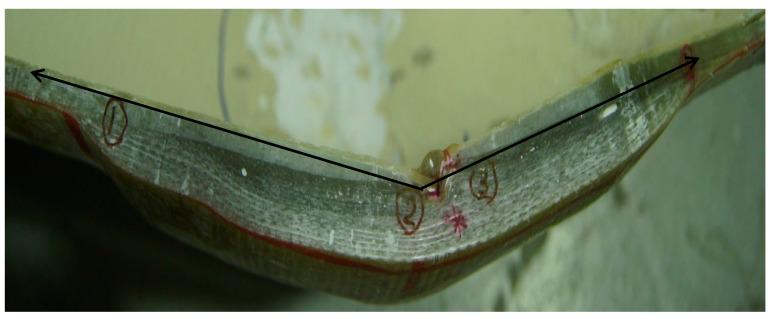
Thickness variation of composite elbow.

For calculations using equation 1a, the miter angle (θ) is idealized as 22.5°. The results indicate that the elbow may withstand the operating pressure depending on the quality factor (E). However, when actual geometric values are input into equation 1b, the allowable pressure calculated is significantly reduced. The result is that the maximum calculated allowable internal pressure when the quality factor E is taken as 1 is only 2 bar. These calculations show that the component’s irregular geometry severely affects the elbow’s load-carrying capacity. The irregularity appears to have contributed directly to the elbow’s premature failure. This is because the elbow wall thickness significantly dominates the axial stress at the center of an elbow. Therefore, it is an inferior design to make the elbow center the thinnest part, as shown in Figure 14. The analytical analysis conclusion is evident in the fact that the elbow was not designed correctly. 

Moisture fiber content influences the degree of crystalline orientation, crystallinity, tensile strength, the porosity of fibers, and swelling behavior. It also lowers the matrix’s glass transition temperature, and in return the composite is degraded due to the softening of the matrix [30,31,32,33,34]. Therefore, one can observe that the interfacial bonding between the matrix and the glass fiber is insufficient since the resin does not impregnate the fiber tows and wet all the fibers. It is well-known that weight gains of 4% are unusual, that those generating significant strains in the 2 and 3 directions, and that restraining them leads to enormous moisture-induced stresses.

## 8. Numerical Analyses

**Figure 15:**
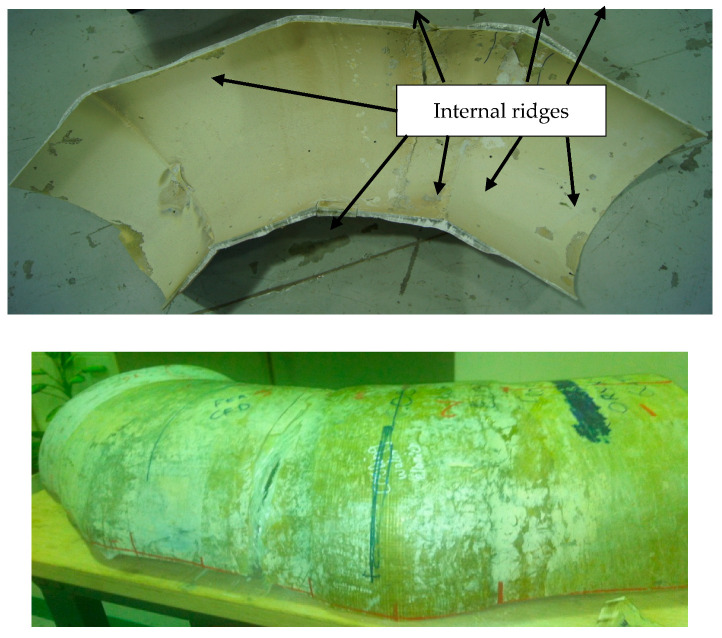
Internal surface of GFRP elbow showing internal ridges.

To further investigate the component’s pressure capacity, it was modeled and analyzed using computational fluid dynamics and finite element analysis techniques. Geometry drawing was performed using Solid Works, and the CFD and FEA analysis was completed using Ansys Workbench. The GFRP elbow dimensions and detailed coordinates were measured using the 3D Computerized Measuring Machine (CMM). Measurements taken included section lengths, bend angles, inner diameter, and wall thickness. These dimensions were then used to draw the real geometry to model the GFRP elbow for numerical evaluation using computational fluid dynamics and finite element analysis. The failure zone was carefully modeled using a fine mesh to accurately compute the pressure profile and stress–strain contours. The measured outer dimensions showed a significant variation through all the directions. The dimensions at the inner side of the failure zone were carefully measured to examine any erosion possibilities. Dimensional analysis eliminated erosion and proved the presence of tiny matrix cracks. It is also interesting to note that based on the developed geometrical profile, the GFRP elbow has a poor profile that includes many internal ridges, as shown in Figure 15. A measured failure zone thickness was 8.3 mm, as highlighted in Figure 14.

The failure analysis includes the effect of fiber–matrix debonding and impact. The computational fluid dynamics and the finite element indicates the following:

- Variable-flowing water continuously eroded and debonded the matrix from the fibers at the innermost layer.
- The combined effect of fiber–matrix debonding and impact accelerated matrix removal at the innermost layer, which initiated the crack.
- As pressure fluctuated inside the elbow pipe, the initial cracks in the weakest location started to grow until they reached the outer surface, which resulted in the rupture of the pipeline at the elbow pipe location.

**Figure 16:**
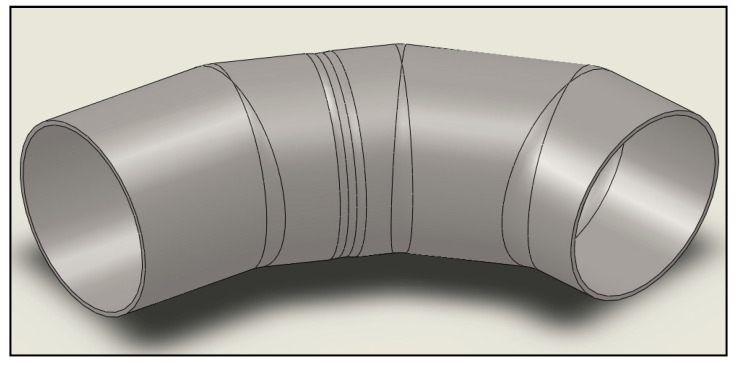
Geometrical modeling.

**Figure 17:**
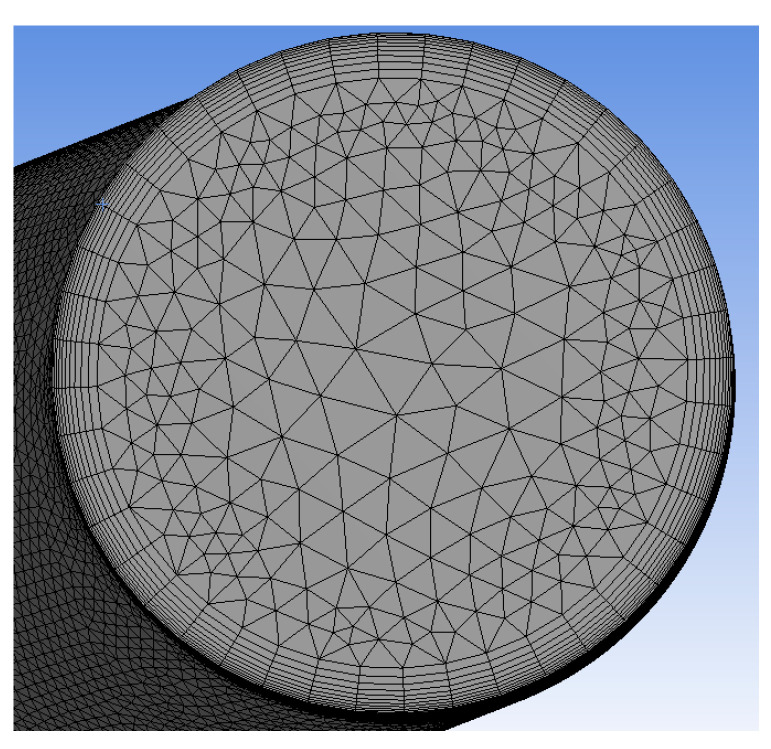
Mesh of fluid zone. Notice the refined mesh near the boundary.

To ensure that the flow is fully developed in the area of interest when analyzed using CFD, 1-m long straight pipe sections were added at the bend’s inlet and outlet. Finally, the pipe’s fluid area was modeled as a solid body for meshing purposes. The developed model is shown in Figure 16a. After the geometric model was completed, it was imported into ANSYS Workbench. The geometry was checked using the ANSYS design modeling tool to ensure that it was appropriately imported and that no errors had occurred. The geometry was then transferred to the meshing tool to mesh. The primary area of interest was the fluid–solid contact area in the elbow. The aim was to refine the mesh in this area to obtain accurate results from the CFD analysis while increasing the mesh size in areas other than interest. This meshing process gives the best mix of quality and speed of analysis. The mesh refinement is achieved using the inflation tool, which reduces the element size near the boundary. The created mesh is shown in Figure 17. 

**Figure 18:**
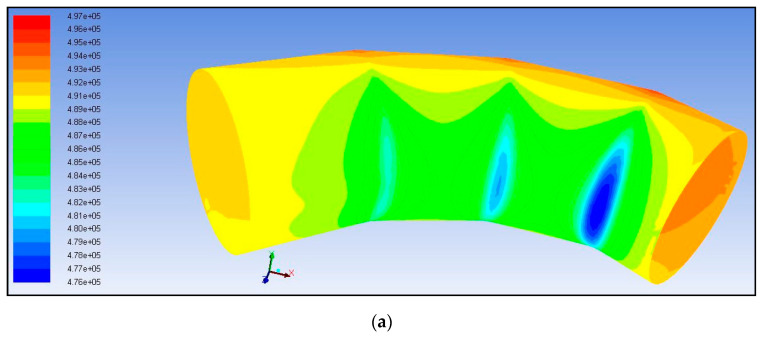

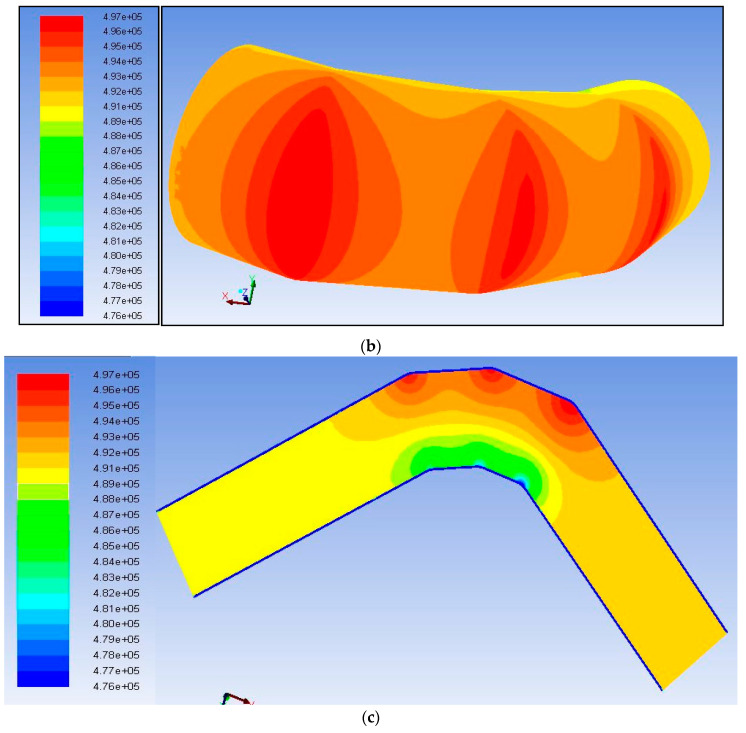
Contours of pressure obtained from CFD analysis.

**Figure 19:**
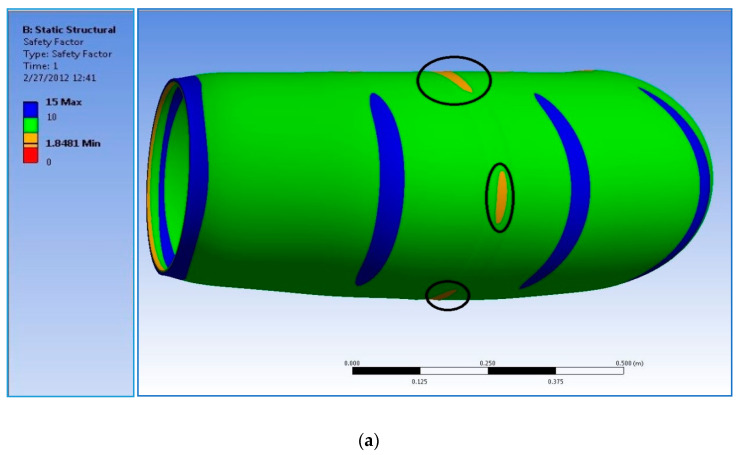

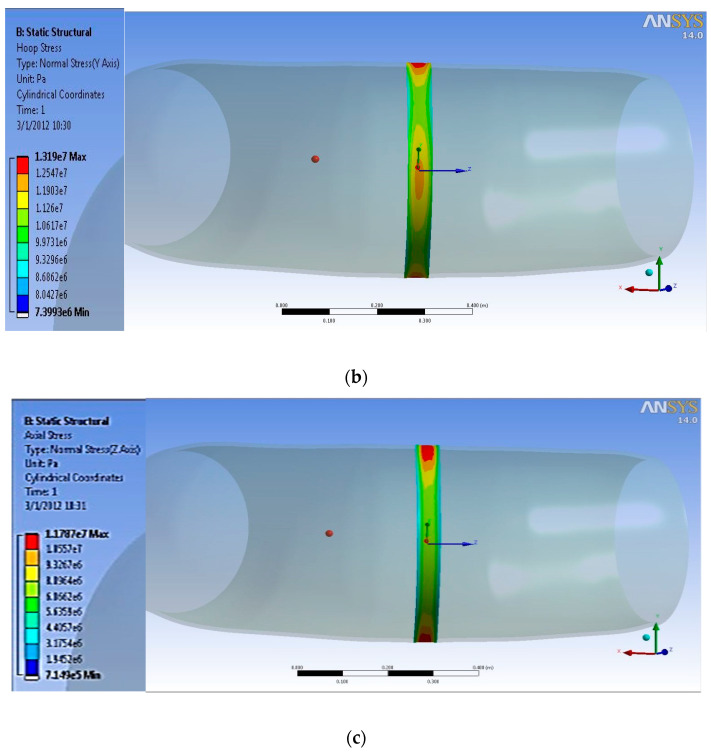
Contours of static safety factor and hoop stress and axial stress at the center of the elbow-joint.

The final mesh was transferred to FLUENT CFD software for analysis. The realizable K-Epsilon model was used to solve the model with a pressure drop of 0.1 bar applied (i.e., 5 bar at the inlet, 4.9 bar at the outlet). The solution was then initialized and iterated until residual values dropped below 1E-3, when they are considered converged for this analysis. After the solution converged, the results could be plotted. The main area of interest was the magnitudes of pressure at the wall. The contours of this pressure were plotted, and the results at the inside of the bend and the outside of the bend are shown in Figure 18a,b, respectively. As seen in Figure 18a, a substantial pressure drop occurs at the first change in direction. This pressure drop causes inefficiency and adds unnecessary head loss in the system, and highlights the component’s geometric irregularity. Using the Ansys Fluid Solid Interaction system, the CFD analysis’s pressure results can be ported into the FEA analysis to study its effect on the material. The data obtained from mechanical testing of the specimens was combined with properties common to glass fiber composites, and was modeled in Ansys. The internal pressure load was imported from FLUENT; fixed supports were applied at either end of the elbow. After the solution was completed, the safety factor’s contours were plotted to determine the component’s ability to withstand the applied pressure load of 5 bar. The results can be seen below in Figure 19. As can be noted from observing the figure, the safety factor dropped to a minimum of 1.8 in the radial region, where the thickness was reduced to simulate the failure zone. A static safety factor of only 1.8 is not enough to give the elbow a lifetime of 20 years under the operating load applied.

## 9. Discussion

When composite materials are loaded in the fiber orientation direction, they will exhibit the best mechanical properties [12]. The fiber-reinforced composites must be able to withstand tensile load pull out, fiber bridge, fiber–matrix debonding, and matrix crack [13]. It is visualized that the elbow pipe is wound with the fiber orientation of ±45°. Based on this finding, the generated strain due to the service load is not principally in the axial and hoop direction. In this case, a maximum strain failure theory does allow cracks in the matrix before cracks in the fiber. The mechanical test indicated that the maximum Ultimate Tensile Strength (UTS) and maximum strain are on the hoop direction specimen, whereas the axial direction showed knee at low stress and low strain, which indicates the formation of the first microcrack in the matrix; the bond between the matrix and the fibers breaks at a microlevel. These observations agree with the work published by [13]. It is observed that micromatrix cracking started at the inner surface of the elbow pipes, and the size and quantity of these microcracks increased with time (see Figure 1c).

The analytical analysis results indicated that the elbow might withstand the operating pressure depending on the quality factor. The initial inspection findings and SEM results indicated an elbow-joint improper fabrication process. Hence, the maximum allowable internal pressure in the elbow is less than the operating pressure due to the component’s irregular geometry. As evidence of this, the results of the numerical analyses indicated that the safety factor in withstanding the operating pressure of 5 bar is dropped down in the radial region where the thickness is reduced to simulate the failure zone. It can be deduced that the leakage initiated when the microcracking and crack coalescence reached the point at which the pressurized water can penetrate through these microcracks to reach the surface of the pipe as surface wetting. Herein, it is worth mentioning that a significant environmental factor that affects the lifetime of the pipe is the moisture content. Moisture content creates swelling stresses—the coefficients of thermal expansion change with temperature. The environmental effects also change the mechanical properties of the material. A leakage path is formed due to microcracking and coalescence from the inner surface towards the outer surface. After many cycles, the pipes reach the point where microcracks turn into microcracks that allow excessive water leakage. The water penetrates through the matrix cracks and propagates them at each cycle, and the cracks coalesce quickly and turn the slight leakage into excessive leakage. At the progressive cycles, it can be concluded that these have propagated and formed all over the elbow pipe surface. Moreover, the ultimate failure occurs with fiber pull-out, delamination between the layers, and fibers’ fracture, as shown in Figure 3. Figure 11 demonstrates the increase in hoop specimens’ delamination area under tension and compression loading state.

## 10. Conclusions

After examining the fabrication process and quality of the elbow in correlation with data obtained from the evaluation techniques, it can be concluded that the following factors contributed to the failure:

- The elbow-joint was not correctly designed and was carelessly and poorly manufactured.
- The fiber reinforcement was E-glass and S-glass, provided by the material datasheet.
- The fiber–matrix interface was poor.
- Irregular thickness: the joints between the straight pipe sections received a more significant amount of reinforcement. This increase in thickness caused the areas of lesser thickness to become inherent weak spots.
- Although the investigated GFRP elbow is not proper, the manufacturer’s specification indicates a minimum allowable thickness of 5.3 mm on the straight pipe. While this may be adequate for straight pipe sections, it is not enough to resist the elbow’s stress intensification.
- It is recommended that sections with a thickness of less than 15 mm be reinforced using overwrapped composites. During the remedy process, enough tension must be applied to the overwrapped composites to achieve a better wetting of fibers by the resin. It is well-known that high fiber tension levels in the fiber-reinforced pipes increase the load-carrying capacity and the stiffness of composite pipes.
- For future installations, it is recommended that the fabrication process is controlled to ensure adherence to standards relating to the glass/resin ratio and proper layup process. The use of ±45° fiber orientation does not provide adequate axial reinforcement.
- The use of multiple layers of chopped strand mat glass fiber would result in a component more suitable to withstand the applied loads for the elbow’s design life.

## Figures and Tables

**Table 1:** Summary of technical details for the composite mitered elbow.

General Information		
**Component Type**	S Glass reinforced polyester resin matrix	
**Process Type**	Cooling water	
**Location**	2.5 m underground	
**Fabrication Process**	Hand layup	
**Service Condition**		
**Operating condition**	Design	Actual
**Operating Temperature (°C)**	5–60	15–36
**Operating Pressure (Bar)**	0.5–7	Up to 5
**Service Life (years)**	20	6
**Mechanical Properties of Polyester resin**		
**Young modulus (GPa)**	3.45	
**Shear modulus (GPa)**	1.30	
**Poisson’s ratio**	0.33	
**Tensile strength (MPa)**	76.0	
**Compressive strength (MPa)**	129.0	
**Density (g/cm^3^)**	2.46	

**Table 2:** Chemical analysis results of the composite elbow specimens.

Element	Actual % (by Weight)		Composition of Glass Fiber Used for Water Transportation		
	Composite Overwrapped	Straight Pipe	E-Glass	C-Glass	S-Glass
SiO_2_	42.2323	22.4456	52.4	64.4	64.4
P_2_O_5_	3.1143	NA	NS	NS	NS
SO_3_	1.5653	5.49190	NS	NS	NS
K_2_O	0.9113	2.63000	0.8	9.6	0.3
CaO	48.4849	62.2219	17.2	13.4	NS
TiO_2_	1.4292	1.04300	NS	NS	NS
Fe_2_O_3_	1.0174	3.1318	14.4	4.1	25.0
BaO	0.2268	NA	NS	0.9	NS

NS: Not specified. NA: Not Available.

## Data Availability

Some or all data, models, or codes that support the findings of this study are available from the corresponding author upon reasonable request.
